# Oridonin ameliorates neuropathological changes and behavioural deficits in a mouse model of cerebral amyloidosis

**DOI:** 10.1111/jcmm.12124

**Published:** 2013-09-05

**Authors:** Zhi-Yuan Zhang, Rolf Daniels, Hermann J Schluesener

**Affiliations:** a Division of Immunopathology of the Nervous System, Institute of Pathology and Neuropathology, University of TuebingenTuebingen, Germany; b Department of Pharmaceutical Technology, University of TuebingenTuebingen, Germany

**Keywords:** Alzheimer’s disease, APP/PS1 transgenic mouse, oridonin, nanoemulsion, cerebral amyloidosis, neuroinflammation, non-mnemonic behaviour

## Abstract

Alzheimer’s disease (AD) is the most common form of neurodegeneration and the major cause of dementia. This multifactorial disorder is clinically defined by progressive behavioural and cognitive deficits, and neuropathologically characterized by β-amyloid aggregation, hyperphosphorylated tau and neuroinflammation. Oridonin, a diterpenoid isolated from Chinese herb Rabdosia rubescens, has multiple biological properties, especially anti-inflammatory and neuroregulatory activities. Potential therapeutic effects of Oridonin were investigated in an animal model of cerebral amyloidosis for AD, transgenic APP/PS1 mice. Oridonin was suspended in carboxymethylcellulose or loaded with a nanostructured emulsion, and was orally administrated or injected. Before, during and following the experimental treatments, behavioural tests were performed with these transgenic mice and their naive littermates. Following relatively short-term treatments of 10 days, brain tissue of mice were removed for immunohistochemical assays. The results indicate that both oral treatment and injection of Oridonin significantly attenuated β-amyloid deposition, plaque-associated APP expression and microglial activation in brain of transgenic mice. Furthermore, injection of Oridonin-nanoemulsion ameliorated deficits in nesting, an important affiliative behaviour, and in social interaction. Additional *in vitro* studies indicated that Oridonin effectively attenuated inflammatory reaction of macrophage and microglial cell lines. Our results suggest that Oridonin might be considered a promising therapeutic option for human AD or other neurodegenerative diseases.

## Introduction

Alzheimer’s disease (AD) is the most common form of neurodegeneration and the major cause of dementia. Alzheimer’s disease is clinically defined by distinct behavioural and cognitive deficits, and is neuropathologically characterized by typical extracellular deposits of aggregated β-amyloid (Aβ) peptide (amyloid plaques).

β-amyloid peptides are toxic and causative in AD, and contribute to memory loss and neurodegenerative pathology 1. Besides, other mechanisms, such as neuroinflammation, play important roles in the pathophysiology of this multifactorial disorder 2. Neuroinflammation is characterized by release of numerous inflammatory mediators and glial activation 3–4. Neuroinflammation may contribute independently to neural dysfunction and cell death, establishing a self-perpetuating vicious cycle by which inflammation induces further neurodegeneration 5. The understanding of neuroinflammation leads to a concept that anti-inflammatory agents may have beneficial effects on neurodegenerative disorders. Indeed, several anti-inflammatories markedly reduced the risk of suffering from this neurologic condition, delayed its onset, ameliorated the symptomatic severity and improved/slowed cognitive decline of AD patients 5.

**Figure 1:**
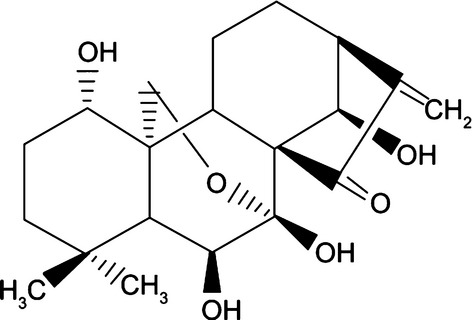
Molecular structure of Oridonin.

Some terpenoids such as ginsenosides, gingkolides and canabinoids are suggested as potential anti-AD agents, as they exhibit promising *in vitro* and *in vivo* biological activities 6. Further studies also suggested potential therapeutic application of diterpenoids for neurogenerative disorders 7,8. Oridonin, a natural diterpenoid compound (Fig. 1) 10, isolated from Chinese herb Rabdosia rubescens, exhibits a variety of biological properties: anti-bacterial, oxygen free-radical scavenging, anti-mutagenic and remarkable anti-neoplastic activities 11–12. Recently, anti-neuroinflammatory and neuroregulatory effects have been reported or suggested by several *in vitro* studies 13,14, which may suggest its potential application against neuroinflammatory and neurodegenerative disorders.

In this study, we used a APP/PS1-21 double transgenic mouse model on a C57BL/6J genetic background that co-expresses the KM670/671NL mutated human amyloid precursor protein and the L166P mutated human presenilin 1 (APP/PS1-21 mice). This mouse model exhibits very aggressive AD pathology, accompanied by neuroinflammation and impairment of cognitive function 16–17. Our aim here was to study potential therapeutic effect of Oridonin on this APP/PS1 mouse model.

## Materials and methods

### Animals

Male APP/PS1-21 mice were obtained from Prof. Jucker (Hertie-Institute, Tuebingen, Germany). Heterozygous male APP/PS1-21 mice were bred with wild-type C57BL/6J females (Charles River Germany, Sulzfeld, Germany). Offspring were tail snipped and genotyped using PCR with primers specific for the APP-sequence (Forward: “GAATTCCGACATGACTCAGG”, Reverse: “GTTCTGCTGCATCTTGGACA”). All experiments were licensed according to the The German Animal Welfare Act (TierSchG) of 2006.

### Materials

Oridonin (>99%) was purchased from Carbosynth Ltd. (Compton, Berkshire, UK). For oral treatment, Oridonin was suspended in 1% carboxymethylcellulose (CMC, Blanose®, Hercules-Aqualon, Düsseldorf, Germany) at a concentration of 2 mg/ml. For injection, Oridonin was loaded with a nanostructured carrier, Lipofundin® (MCT, 10% for infusion, B. Braun AG, Melsungen, Germany), by high pressure at a concentration ratio of 2 mg/ml. A quantity of 30 mg Oridonin was first coarsely dispersed in 60 ml Lipofundin. Subsequently, the dispersion was high-pressure homogenized using an Emulsiflex C3 (Avestin Inc., Canada). At first five cycles at 750 bar and then five cycles at 1750 bar were run yielding ∼50 ml of a 2 mg/ml formulation.

### Treatment with Oridonin

Six groups of animals (*n* = 6, three males and three females) were grouped for two experimental treatments: daily oral administration with Oridonin suspension (20 mg/kg bodyweight) at the age of 5 months and daily i.p. injection with Oridonin-nanoemulsion (20 mg/kg bodyweight) at the age of 3 months. Each experiment lasted 10 days and contained three groups: Oridonin treatment, vehicle control and control without any treatment.

### Design and evaluation of nest construction assay

A nest construction assay 18 was modified to determine the deficits in affiliative/social behaviour of these APP/PS1 mice and potential changes following treatment.

Mice were individually housed for at least 24 hrs in clean plastic cages with ∼1 cm of wood chip bedding. Two hours prior to the onset of the dark phase, individual cages were supplied with paper towel torn into ∼5 × 5 cm squared pieces. The next morning, cages were inspected for nest construction. Paper towel nest construction was scored by a three-point system: 1 = no biting or tears on the paper, 2 = moderate biting and/or tears on the paper but no coherent nest and 3 = the vast majority of paper torn into pieces and grouped into a corner of the cage 18.

### Social interaction assay

The social interaction assay was performed according to previous studies 19–20 with minor modifications. The social interaction assay was video recorded to quantify all distinct behaviours of naive mice, vehicle-treated and Oridonin-treated APP/PS1 mice as ‘resident’ in the presence and absence of an ‘intruder’ mouse. Their movements were then analysed to assess overall activity level and overt neurological differences. Individual mice were placed in a clean plastic housing cage identical with the home cage for 15 min. to establish each as the ‘resident’ mouse. An age-, weight- and gender-matched non-treated naive mouse was introduced as the ‘intruder’ for a second 15 min. period. Both 15 min. sessions were video recorded, all identifiable distinct independent and interactive behavioural events 19–20, for the resident mouse (15 min. alone; 15 min. with intruder) and for the intruder mouse (15 min. with resident), were counted by three independent observers blinded to treatment categories.

### Immunohistochemistry and image evaluation/analysis

Oridonin-treated and control mice were killed after 10-days-treatment. They were perfused intracardially with 4°C, 4% paraformaldehyde in PBS. Brains were quickly removed and post-fixed in 4% paraformaldehyde overnight at 4°C. Brains were then cut into two hemispheres, embedded in paraffin, serially sectioned (3 μm) and mounted on silan-covered slides. Hemispheres sections were stained by immunohistochemistry as described previously 21, with following antibodies: β-amyloid (1:100; Abcam, Cambridge, UK) for Aβ deposition, Iba-1 (1:200; Wako, Neuss, Germany) for activated microglia, GFAP [1:500; Chemicon (Millipore), Billerica, MA, USA] for astrocytes and anti-Alzheimer precursor protein A4 (APP, 1:200, Chemicon International, Temecula, CA, USA). The rabbit polyclonal anti-β-amyloid antibody (ab2539) was generated against the synthetic peptide DAEFRHDSGYEVHH conjugated to KLH, corresponding to amino acids 1-14 of human β-amyloid.

After immunostaining, hemisphere sections were examined and photographed by light microscopy (Nikon Coolscope, Nikon, Düsseldorf, Germany). All sections were randomly numbered and analysed by two observers independently, who were not aware of treatments. β-amyloid plaques, APP plaque-like staining, Iba-1^+^ and GFAP^+^ cells in neocortex and hippocampus were counted. Small Aβ plaques with a dense core or larger plaques with a dense core and a large halo of diffuse amyloid were counted. Small points or spots of Aβ staining, smaller than a cellular nucleus (around 10 μm) and slightly stained diffuse amyloid without a dense core were classified as unclear deposition and were not counted. APP staining was located on cells or outside cells presenting a plaque-like expression pattern. The numbers of APP ‘plaques’ were also counted, according to the standard of Aβ plaques. Further, images of hemisphere cross-sections were captured with fixed parameters; the neocortex and hippocampus on the images were outlined and analysed using the software MetaMorph Offline 7.1 (Molecular Devices, Toronto, ON, Canada). Area percentages of specific immunoreactivity (IR) were selected by colour threshold segmentation and calculated. Results were given as arithmetic means of plaque/cell counts or area percentages of IR to interest areas on cross-sections and SEM.

### *In vitro* assays

The immortalized murine macrophage cell line RAW 264.7 and microglia cell line N9 were used to determine effects of Oridonin on inflammatory reaction of macrophages and microglia *in vitro*. RAW 264.7 cells and N9 were seeded into 12-well cell culture plates and cultured for 24 hrs. Afterwards, cells were stimulated with lipopolysaccharide (LPS; 1 μg/ml), and incubated with or without Oridonin (1 μg/ml, dissolved in media) for 24 and 48 hrs. Thereafter, supernatants were collected for analysis of nitric oxide concentration by standard Griess assay (Sigma-Aldrich, Munich, Germany), and cells were harvested and collected. Total RNA from cultured cells was prepared, reverse transcribed into cDNA and subjected to subsequent semi-quantified PCR analysis using primers specific for mouse iNOS, IL-1β, IL-6 and β-actin. Results were calculated as levels of target mRNAs relative to those of β-actin (*n* = 3). In addition, cell viability was detected by MTT colorimetric assay and determined by analysis of cellular confluent and morphology, using microphotos and the software MetaMorph.

### Statistical analysis

Differences of plaque/cell counts, area percentages, behavioural data or mRNA expression levels between treatments and controls were analysed by unpaired *t*-tests (Graph Pad Prism 5.0 software). For all statistical analyses, significance levels were set at *P* < 0.05.

## Results

### Effects of oral Oridonin treatment on neuropathological changes and neuroinflammation

Amyloid plaques were distributed throughout the whole cortex of 5 months old APP/PS1 transgenic mice, some of them were small, dense core plaques and some were larger plaques with a dense core and a large halo of diffuse amyloid. In hippocampus, obviously lower plaque density was seen.

**Figure 2:**
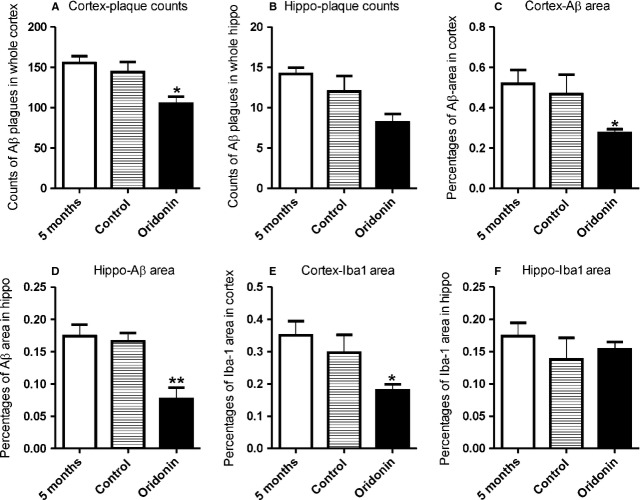
Oridonin reduced β-amyloid (Aβ) deposition and microglial activation. APP/PS1 mice at 5 months of age received Oridonin or vehicle (CMC, as control) by gavage for 10 days. Brains of these mice and of another group of 5-month-old untreated littermates were analysed by immunohistochemistry. Arithmetic means of IR area percentages and of plaque/cell counts from different treatment groups are represented in the bar graphs. Differences of plaque/cell counts or area percentages between treatment and control were analysed by unpaired *t*-test (Graph Pad Prism 5.0 software, significance levels were set at *P* < 0.05). In both, cortex and hippocampus, of APP/PS1 mice from the control group (vehicle) and their age-/gender-matched littermates (received no treatment, noted as ‘5 month’), no significant difference in Aβ deposition (in terms of plaque counts and IR area) and Iba-1 expression was observed. (A) In the cortex of APP/PS1 mouse brains from the Oridonin group, significantly reduced numbers of amyloid plaques were counted. (B) In hippocampus, however, no significant reduction of plaque counts was observed. (C and D) IR area percentages of Aβ staining were dramatically reduced in both cortex and hippocampus following Oridonin treatment. (E and F) Following Oridonin treatment, Iba-1 IR was significantly reduced in the cortex, but not in hippocampus.

**Figure 3:**
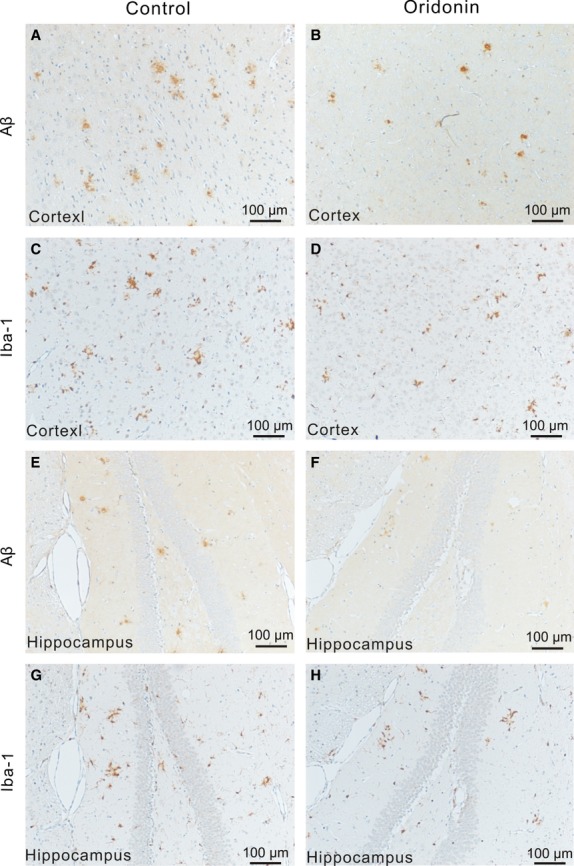
Therapeutic effect of Oridonin on β-amyloid (Aβ) deposition and microglial activation. Representative microimages show the changes in Aβ deposition and microglial activation following Oridonin treatment. (A and B) In the cortex of APP/PS1 mice from the control group (A), more and relatively larger-sized Aβ plaques were observed, compared with the Oridonin group. (C and D) Iba-1 staining showed more counts and larger IR area of Iba-1^+^ cells in cortex from the control group (C), most Iba-1^+^ microglia accumulated surrounding Aβ plaques (according to the serial sections to the sections of Aβ staining (A). (E and F) In hippocampus from the control group (E), more and slightly larger-sized Aβ deposition can be seen, compared with the Oridonin treatment group (F). (G and H) Iba-1 staining on serial sections (to the sections of Aβ staining) showed that most Iba-1^+^ microglia were clustered around Aβ plaques, but there are also numerous Iba-1^+^ cells distributed throughout the hippocampus, in both control and Oridonin treatment groups.

No significant difference of Aβ deposition or of Iba-1 expression in both cortex and hippocampus could be observed between APP/PS1 mice from the control group (vehicle only) and their littermates, which received no treatment and were noted as ‘5 months’ in the figures. The oral treatment with Oridonin attenuated neuropathological changes, compared to the age- and gender-matched control mice. The Oridonin treatment reduced the plaque counts significantly in cortex (control = 144.2 ± 12.4, Oridonin = 104.8 ± 8.8, *P* < 0.05, *n* = 6) and slightly in hippocampus (control = 12.0 ± 1.9, Oridonin = 8.2 ± 1.1, *P* > 0.05, *n* = 6). It significantly decreased the Aβ IR area in both cortex and hippocampus (cortex: control = 0.47 ± 0.10%, Oridonin = 0.28 ± 0.02%, *P* < 0.05; hippocampus: control = 0.17 ± 0.01%, Oridonin = 0.08 ± 0.02%, *P* < 0.01, *n* = 6; Fig. 2). Further, in the Oridonin-treated group, Aβ plaques had a smaller size and fewer branches (Fig. 3).

**Figure 4:**
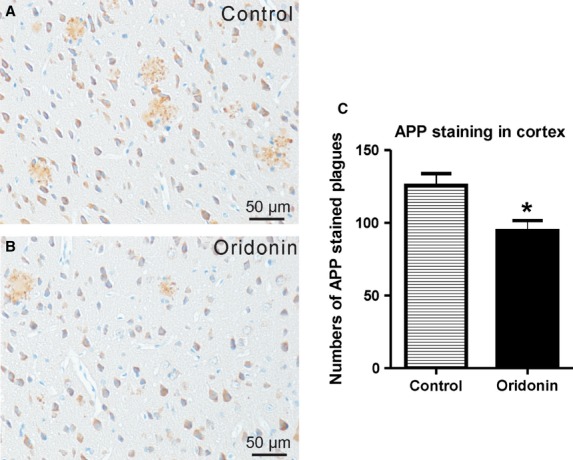
Therapeutic effect of Oridonin on APP expression. APP/PS1 mice at 5 months of age received Oridonin or vehicle by gavage for 10 days. Brains of these mice were analysed for APP expression by immunohistochemistry. (A and B) The APP staining is mainly located on neurons and in/around plaques presenting a plaque-like expression pattern, as presented in the figure. Representative microimages A and B show the changes in plaque-associated APP expression following Oridonin treatment. (C) Arithmetic means of APP plaque numbers from treatment and control groups are represented in the bar graphs. The numbers of plaques covered or surrounded by APP staining are significantly reduced in cortex. Differences of APP plaque counts between treatment and control were analysed by unpaired *t*-test (Graph Pad Prism 5.0 software). For all statistical analyses, significance levels were set at *P* < 0.05.

APP expression in cortex and hippocampus was further investigated. The APP staining was mainly located on neurons and in/around plaques presenting a plaque-like expression pattern, as presented in the Figure 4A and B. Total areas of APP staining in cortex or hippocampus were not significantly changed after the treatment. However, the numbers of plaques covered or surrounded by APP staining were significantly reduced in cortex, but not in hippocampus (in cortex: control = 125.7 ± 8.2, Oridonin = 94.5 ± 7.0, *P* < 0.05, *n* = 6; Fig. 4C). APP staining associated with plaques in the cortex could not be quantitatively evaluated. Similar results of APP expression were obtained from the treatment with nanoemulsion injections (data not shown).

In both cortex and hippocampus, amoeboid Iba1-positive microglia were observed clustered around amyloid deposits (Fig. 3). Oridonin treatment significantly reduced the IR area of Iba-1 in cortex (control = 0.30 ± 0.05%, Oridonin = 0.18 ± 0.02%, *P* < 0.05, *n* = 6), but not in hippocampus (control = 0.14 ± 0.03%, Oridonin = 0.15 ± 0.01%, *P* > 0.05, *n* = 6; Fig. 2). There were fewer cells expressing Iba-1, major part of these Iba-1^+^ cells were also less clustered around plaques in cortex following Oridonin treatment, indicating reduced microglial activation (Fig. 3).

Numerous GFAP^+^ cells (GFAP-IR) were widely distributed throughout the hippocampus and cortex. They all showed typical morphology of astrocytes, including stellate shape and multiple branched processes. No significant changes in GFAP staining (IR area) were observed between Oridonin treatment and controls (data not shown).

### Effect of Oridonin-nanoemulsion injection on Aβ deposition and microglial activation

**Figure 5:**
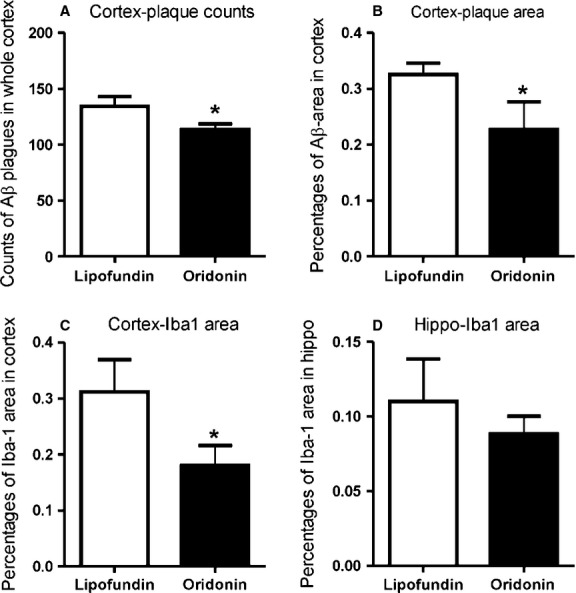
Oridonin-nanoemulsion (injection) reduced β-amyloid (Aβ) deposition and microglial activation mainly in cortex. APP/PS1 mice at 3 months of age received daily i.p. injection of Oridonin-nanoemulsion or vehicle (Lipofundin) for 10 days. Arithmetic means of IR area percentages or plaque numbers from different groups are represented in the bar graphs. Differences of plaque/cell numbers or area percentages between treatment and control were analysed by unpaired *t*-test (Graph Pad Prism 5.0 software). For all statistical analyses, significance levels were set at *P* < 0.05. (A and B) In the cortex of APP/PS1 mice following Oridonin-nanoemulsion injection, numbers of amyloid plaques were significantly reduced, as well as area percentages of Aβ deposition (positive pixels), compared with the control group. (C) In cortex, Iba-1 IR was reduced by nearly half in the Oridonin treatment group compared with the controls. (D) However, only slightly decreased Iba-1 IR was observed in hippocampus.

There was also no significant difference in Aβ deposition between the control group (vehicle only) and their littermates receiving no treatment, for the injection experiment at the age of 3 months. In hippocampus, from both Oridonin and control groups, Aβ deposition could be barely seen, which was in accordance with the original report that amyloid plaques appeared first in the cortex at 6 weeks of age and rather later in the hippocampus of APP/PS1 transgenic mice 17. In the cortex of APP/PS1 mice from the Oridonin group, significantly reduced numbers of amyloid plaques were counted (control = 134.2 ± 8.8; Oridonin = 113.5 ± 5.1; *P* < 0.05, *n* = 6; Fig. 5). Area percentages of Aβ deposition (IR) were significantly reduced in the cortex as well (control = 0.33 ± 0.02%; Oridonin = 0.23 ± 0.05%; *P* < 0.05, *n* = 6; Fig. 5). Many plaques of smaller size were observed in the brains from the Oridonin-nanoemulsion group, therefore stronger reduction could be observed in area of Aβ IR.

Numbers of Iba-1^+^ cells in cortex and hippocampus did not show a significant change following Oridonin treatment. However, Iba-1 IR was reduced by nearly the half in cortex (control = 0.31 ± 0.06%, Oridonin = 0.18 ± 0.04%, *P* < 0.05, *n* = 6), but was only slightly decreased in the hippocampus (control = 0.11 ± 0.03%; Oridonin = 0.09 ± 0.01%; *P* > 0.05, *n* = 6; Fig. 5). In cortex from Oridonin-treated mice, major part of Iba-1^+^ cells were less clustered and showed smaller size and less branched morphology.

### Effect of Oridonin-nanoemulsion injection on affiliative behaviour impairment (nest construction assay)

**Figure 6:**
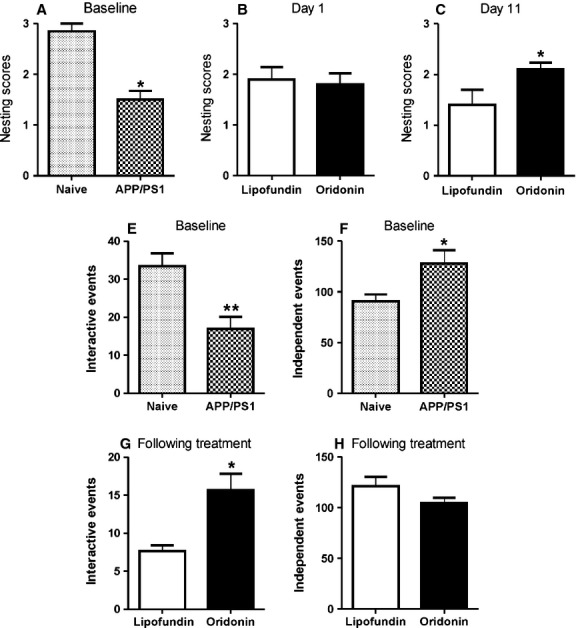
Effect of Oridonin-nanoemulsion (injection) on behavioural impairments (nest construction assay and social interaction). APP/PS1 mice received daily i.p. injection of Oridonin-emulsion or vehicle (Lipofundin, as control) for 10 days. Together with naive mice, they were assessed for nesting behaviour and social interaction. Nest construction was explored with paper towel material using a 3-point scaling system (see Materials and methods) in naive and APP/PS1 mice. Social interaction was determined by a resident-intruder assay (see Materials and methods) (E–H). (A) An impaired nesting ability was observed for APP/PS1 mice. (B) No significant difference between the Oridonin and the control group could be observed right at the beginning of treatment, namely at day 1. (C) A significant difference between Oridonin injection and control groups was observed after 10-days treatment, namely at day 11. (E and F) As baseline controls of social interaction, APP/PS1 mice have less interactive behavioural events and more independent behavioural events together with introduced intruder mice compared with naive mice. (G and H) Following treatment, increased interactive behavioural events were observed in the Oridonin group compared with the controls.

To assess the influence of Oridonin-nanoemulsion injection on affiliative behaviour, we analysed nest construction in naive and APP/PS1 mice. Before treatment, ability of nest construction was compared between APP/PS1 mice and naive mice, and an impaired nesting ability was observed for transgenic mice (naive = 2.9 ± 0.2, APP/PS1 = 1.5 ± 0.2, *P* < 0.05, *n* = 6, Fig. 6A). Right at the beginning of treatment, namely at Day 1, no significant difference between Oridonin-treated and control groups was observed (control = 1.9 ± 0.2, Oridonin = 1.8 ± 0.2, *P* > 0.5, *n* = 6, Fig. 5B). However, a significant difference between treatment and control groups could be observed at Day 11 (control = 1.4 ± 0.3, Oridonin = 2.1 ± 0.1, *P* < 0.05, *n* = 6, Fig. 5C). After the 10-days treatment of Oridonin, relatively immediate chewing and tearing behaviour towards the paper towels were observed; paper towels were torn into pieces and grouped into a corner of the cage. In contrast, APP/PS1 mice from the control group investigated and slightly chewed but did not really destruct the paper towels; paper towels were found all over in the cage, not grouped.

Oral Oridonin treatment improved impaired ability of nest construction of these transgenic mice, but not statistically significantly (data not shown).

### Effect of Oridonin-nanoemulsion injection on social interaction

During the social interation test, no overt differences were observed between any of the transgenic mice. The interactive behaviour of APP/PS1 mice was clearly distinct from naive and Oridonin-treated transgenic mice. Resident APP/PS1 mice showed a significantly lower frequency of interactive behaviour compared to that of naive mice (naive = 33.3 ± 3.5, APP/PS1 = 17.0 ± 3.1, *P* < 0.05, *n* = 6) and higher frequency of independent behaviour (naive = 90.7 ± 6.6, APP/PS1 = 128.0 ± 13.0, *P* < 0.05, *n* = 6) in the presence of the intruder mouse (Fig. 6E and F). Following the 10-days treatment, Oridonin-nanoemulsion restored this impairment comparable to control levels, namely increased the frequency of interactive behaviour significantly (control = 7.7 ± 0.8; Oridonin = 15.7 ± 2.2; *P* < 0.05, *n* = 6) and decreased the frequency of independent behaviours slightly (control = 121.0 ± 9.2; Oridonin = 104.5 ± 5.4; *P*> 0.05, *n* = 6; Fig. 6G and H).

### Oridonin attenuated inflammatory activation of macrophages and microglia cell lines *in vitro*

**Figure 7:**
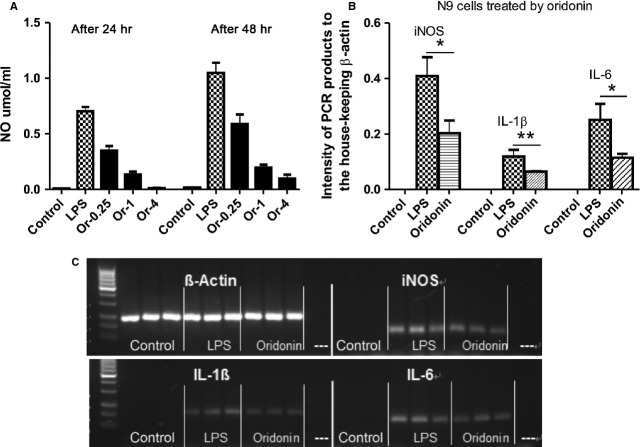
Anti-inflammatory activity of Oridonin in microglia cell culture. Effects of Oridonin on microglia/macrophage activation were analysed *in vitro* using murine microglia cell line N9. Cells were seeded into 12-well cell culture plates and cultured for 24 hrs. Afterwards, cells were stimulated with lipopolysaccharide (LPS, 1 μg/ml), and incubated with or without Oridonin (1 μg/ml) for 24 or 48 hrs. (A) Supernatants were collected for the analysis of nitric oxide concentrations by a standard Griess assay. (B) Bar graphs show semi-quantified results of imaging intensity relative to housekeeping gene β-actin. Total RNA from cultured cells was prepared and mRNA levels of iNOS, IL-1β and IL-6 was measured by PCR. Results were calculated as levels of target mRNAs relative to those of β-actin (three samples from each group). The unpaired *t*-test was performed to compare differences between control and Oridonin treatment (Graph Pad Prism 5.0 for Windows). *P < 0.05, **P < 0.01 compared with their respective LPS alone group. (C) Photos of gel electrophoresis with PCR products show reduced mRNA levels of iNOS, IL-1β and IL-6 after treatment by Oridonin.

In addition, effects of Oridonin on inflammatory activation of macrophage and microglia *in vitro* using murine macrophage cell line RAW 264.7 and murine microglia cell line N9 were studied. Inflammatory macrophage activation was induced by LPS (1 μg/ml); with or without Oridonin treatment for 24 and 48 hrs. Following LPS induction significantly increased nitric oxide production and mRNA expression of iNOS, IL-1β and IL-6 indicated an inflammatory activation. Oridonin significantly reduced the nitric oxide concentration and attenuated mRNA expression of iNOS, IL-1β and IL-6, suggesting an effective anti-inflammatory activity of Oridonin for both macrophage and microglia cell lines. Oridonin had very similar effects for N9 and RAW cell cultures, results from N9 cell culture are shown in the Figure 7. In addition, cell viability during treatments was proven by MTT assay and morphology analysis (data not shown).

## Discussion

In this study, we describe therapeutically beneficial effects of Oridonin in a rodent model of cerebral amyloidosis. An oral treatment with Oridonin suspension significantly attenuated Aβ deposition, plaque-associated APP expression and microglial activation in both cortex and hippocampus of APP/PS1 mice at 5 months of age. Further, treatment of i.p. injection with Oridonin-nanoemulsion effectively ameliorated neuropathological changes. Interestingly, it also restored impaired nesting behaviour and social interaction.

Terpenoids are promising for treatment of neurodegenerative disorders, especially of AD 6. Furthermore, several potent diterpenoids, such as diterpenoids from the Croton tonkinensis 7, ‘serofendic acid’ derived from foetal calf serum 8 and diterpenoids isolated from Salvia miltiorhiza 9, are neuroprotective, promoting the survival of neurons or have acetylcholinesterase-inhibitory effects. Oridonin possesses anti-bacterial, anti-tumour and anti-inflammatory properties. Several recent studies showed that Oridonin suppressed the expression of iNOS and COX-2 through inhibition of NF-κB DNA binding 22–23; inhibited production of various proinflammatory cytokines, induced apopotosis of immune cells. It as well restored impaired regulatory T cells and Th1/Th2 balance 24. Moreover, Oridonin has been reported to have anti-neuroinflammatory and neuroregulatory effects through modulation of multiple functions of microglia, which have been implicated as culprits in many neurological disorders 15. An important role of neuroinflammation involved in AD pathology has been reported in animal models and human 5. Attenuated neuroinflammation has been shown to contribute to reduced hallmark features of AD pathology, including Aβ-plaque accumulation 25, and therapeutic strategies controlling the activation of microglia and the excessive production of proinflammatory factors may control neurodegeneration in dementia 26. Modulation of nitric oxide levels has been reported in glial cells activated during the neuroinflammatory response. Cytokines such as IL-1β and IL-6 are directly involved in the development of neuroinflammation and neurodegeneration. Oridonin effectively attenuated inflammatory reaction and reduced production of nitric oxide and pro-inflammatory cytokines/molecules in our macrophage and microglia cell cultures. The levels of these cytokines/molecules were reported to correlate with amyloid load in a similar transgenic mouse model of AD 27. Further, Oridonin significantly attenuated the microglial activation in brains of transgenic mice, suggesting an inhibitory effect of Oridonin on neuroinflammation, which may contribute to the ameliorated pathology and improved behaviour.

As a potent inhibitor of NF-κB 23, Oridonin was suggested to be of therapeutic importance for the treatment of AD pathology, not only by blocking inflammatory processes but also by directly inhibiting the production of Aβ peptides 28. Reduced Aβ deposition and decreased plaque-related APP expression observed in our study might therefore directly attribute to NF-κB inhibition and its effect on Aβ peptides production.

Deficits in cognitive and non-mnemonic behaviours are debilitating features of most neurodegenerative diseases of the central nervous system, including AD. Toxic Aβ peptides, especially APP, inflammatory reaction and inflammation markers/molecules are all reported to be directly associated with deficits in cognitive function and behaviour 29. Alzheimer’s disease mouse models are valuable for modelling not only cognitive impairment but also disturbances in non-mnemonic behaviours 30.

Nesting behaviour is an affiliative, social behaviour and important for the survival of animals. Impaired nesting behaviour has been observed in a similar AD rodent model, Tg2576, overexpressing APP 18. In the APP/PS1 model, we observed similar impairment of nesting behaviour compared with the naïve control. The impaired nest construction behaviour was significantly restored by Oridonin-nanoemulsion treatment.

Alzheimer’s disease is also characterized by deficits in social communication and social memory. With a comparable AD model of APPswe/PS1 mice, impairment in social interaction has been reported: while wild-type mice were more willing to explore the stimulus mouse than an empty cage, APPswe/PS1 mice were not 31. A more recent study reported very early (3 months) occurrence of these deficits in comparable transgenic mice and suggested that social deficits precede other neuropsychiatric and cognitive AD-like symptoms and can be employed as early markers of AD pathology in transgenic mouse models 32. In our present study, similar deficits in social interaction of transgenic mice were observed, compared to naive littermates. Treatment with Oridonin-nanoemulsion then significantly ameliorated this impairment. Considering the relatively short term of the treatment, reduced Aβ deposition, decreased APP expression, especially plaque-associated APP density and attenuated neuroinflammatory reaction may contribute to the improved nesting behaviour and social interaction.

Recently, many experimental treatments with anti-inflammatory agents were tested in animal models of AD as well, such as: CHF5074, a non-steroidal anti-inflammatory drug 33; aspirin-triggered LXA4 34; telmisartan 35; cyclooxygenase-1 36; minocycline 37 and several cannabinoids 5. They inhibited or modulated microglial activation, attenuated neuroinflammatory reaction, reduced AD-like pathology, some of them also improved cognitive deficits in transgenic mouse models of AD, similar as Oridonin’s effect observed in our present study. All these together suggest anti-inflammatory agents as a promising therapeutic option for AD.

However, it is possible that additional or other mechanisms than anti-inflammation contribute to effects of Oridonin, such as direct reduction of Aβ peptides production through NF-κB inhibition. Furthermore, Oridonin is not a general immunosuppressant, it not only inhibits overproduction of immune cells and cytokines 13 but also contributes to switch of inflammatory immune cells to regulatory cells, to maintain immune homeostasis 24. Rabdosia rubescens and its aqueous extract including Oridonin have been applied for centuries and have shown good tolerance and safety, also in elderly patients.

Because of poor water solubility and short biological half-time of Oridonin, studies were focusing on nanostructured carriers which can provide better bioavailability, prolonged retention time in blood, improved entrapment efficiency and controlled drug release 12–38. More importantly, nanoemulsions also serve as a safe and effective delivery vehicle across oral and CNS barriers 39. All of these may contribute to an increase in therapeutic effects, which is in accordance with our results, especially on behavioural improvement.

Taken together, treatments with Oridonin, suspended in cellulose or loaded by nanoemulsion, by gavage or per injection, effectively ameliorated neuroinflammatory reaction and cerebral amyloidosis in our transgenic mouse model. Oridonin-nanoemulsion injection further restored impaired affiliative and social interactive behaviours. These behavioural and pathological effects of Oridonin may be because of multiple mechanisms/factors including reduced inflammatory activation of glial cells and immune cells, decreased Aβ deposition and APP expression directly or indirectly, as well as possible neuroregulatory/protective effects through modulated microglial function and reduced local production of proinflammatory factors. All of these results suggest that Oridonin may be considered a promising therapeutic option of human AD or other neurodegenerative disorders.
